# Peripheral *CHI3L1* expression is associated with APOE ε4 status in early-onset Alzheimer’s disease

**DOI:** 10.3389/fnagi.2025.1730319

**Published:** 2025-12-04

**Authors:** Anja Steinmaurer, Lina Breit, Elisabeth Stögmann, Theresa König

**Affiliations:** 1 Department of Neurology, Medical University of Vienna, Vienna, Austria; 2 Comprehensive Center for Clinical Neurosciences and Mental Health, Medical University of Vienna, Vienna, Austria

**Keywords:** Alzheimer’s disease, neuroinflammation, CHI3L1/YKL-40, gene expression, neurological disease, apolipoprotein E, early-onset Alzheimer’s disease, peripheral biomarkers

## Abstract

**Background:**

YKL-40 (*CHI3L1*) is a glycoprotein secreted by reactive astrocytes and peripheral immune cells, implicated in inflammation and tissue remodeling in Alzheimer’s disease (AD). While elevated *CHI3L1* levels have been observed in cerebrospinal fluid and plasma, its expression at the transcript level in peripheral blood - and modulation by genetic risk factors such as *APOE* ε4 - remains poorly understood.

**Methods:**

We analyzed peripheral blood *CHI3L1* mRNA expression in a well-characterized cohort comprising individuals with biomarker-confirmed AD (*n* = 34), mild cognitive impairment (MCI; *n* = 31), and cognitively healthy controls (HC; *n* = 21). *CHI3L1* expression levels were compared across diagnostic groups and stratified by *APOE* ε4 status, age at onset (early-onset < 65 years; late-onset ≥ 65), and sex. Correlations were examined between *CHI3L1* and inflammatory gene transcripts (*IL1B*, *TNF*, *MMP9*, *LRP1*, and *TREM2*).

**Results:**

Peripheral *CHI3L1* expression was elevated in individuals with early-onset AD (EOAD), particularly among *APOE* ε4 carriers (EOAD *APOE* ε4+, *n* = 13 vs. EOAD *APOE* ε4-, *n* = 8; *p* = 0.026). Stratified analyses revealed an exploratory association between *CHI3L1* expression, *APOE* genotype, and sex, with the highest levels observed in female ε4 carriers with EOAD. Across diagnostic groups, *CHI3L1* levels positively correlated with transcripts of *IL1B*, *MMP9*, and *LRP1*, with the strongest associations again in *APOE* ε4 + individuals. Notably, these effects were more pronounced in the MCI and AD groups than in healthy controls, indicating early immune activation in at-risk individuals.

**Conclusion:**

Our exploratory findings indicate that peripheral *CHI3L1* expression may reflect APOE ε4-linked immune activity, with a trend toward higher expression in EOAD and in female ε4 carriers. The observed genotype- and sex-dependent expression patterns indicate preliminary differences in peripheral immune activity that warrant replication in larger cohorts. Peripheral *CHI3L1* may thus serve as a hypothesis-generating marker of genotype-linked inflammatory phenotypes rather than a validated biomarker.

## Background

Alzheimer’s disease (AD) is the leading cause of dementia worldwide, currently affecting over 55 million people - a figure projected to nearly triple by 2050 due to aging populations (Nichols et al., 2022). While its core neuropathological features - including extracellular accumulation of amyloid-beta (Aβ) plaques and intracellular tau neurofibrillary tangles (Nichols et al., 2022) - have been well characterized, accumulating evidence implicates inflammatory mechanisms as a relevant contributor to disease pathogenesis, extending beyond the central nervous system (CNS) to include systemic immune processes (Walker et al., 2019). The apolipoprotein E (*APOE*) ε4 allele represents the strongest genetic risk factor for sporadic AD, traditionally linked to earlier age at onset (AAO), increased amyloid burden, and enhanced neuroinflammation. However, increasing research indicates a broader role for *APOE* ε4 in the regulation of immune function. Specifically, *APOE* genotype has been shown to influence both central neuroinflammatory responses, including microglial activation, and peripheral immune activity, suggesting that *APOE*-dependent modulation of inflammation may contribute to disease heterogeneity (Kloske and Wilcock, 2020).

YKL-40 (chitinase-3-like protein 1, *CHI3L1*) is a glycoprotein involved in extracellular matrix remodeling, immune modulation, and tissue repair. It is of particular interest in disease-related inflammatory processes, being expressed in immune cells of both the peripheral and CNS compartments. In the CNS, YKL-40 is predominantly expressed by reactive astrocytes and activated microglia. Elevated YKL-40 levels have been consistently reported in the cerebrospinal fluid (CSF) and plasma of individuals with AD, correlating with tau pathology, neuronal injury, and clinical progression (Bonneh-Barkay et al., 2010; Kester et al., 2015; Llorens et al., 2017; Pase et al., 2024). Moreover, recent clinical evidence has linked serum YKL-40 levels to increased risk of recurrent intracerebral hemorrhage in patients with cerebral amyloid angiopathy (CAA), independent of age, hemorrhage volume, and MRI markers of small vessel disease (Xu et al., 2024). Peripherally, YKL-40 is produced by monocytes and macrophages, and has been implicated in systemic inflammatory conditions, including cardiovascular and metabolic diseases (Deng et al., 2020). These findings suggest that YKL-40 may act not only as a marker of glial activation but also as an active mediator of inflammation across biological compartments.

Despite growing evidence for YKL-40’s role in both central and peripheral inflammatory processes, its expression at the mRNA level in peripheral blood and its modulation by genetic factors such as *APOE* ε4 remain poorly characterized. Unlike protein measurements that reflect cumulative secretion, peripheral *CHI3L1* mRNA captures upstream and can identify early immune signaling events. Assessing *CHI3L1* mRNA expression may therefore reveal genotype-linked immune patterns not evident from protein assays.

In this exploratory study, we investigated peripheral *CHI3L1* mRNA expression in biomarker-confirmed individuals with AD, mild cognitive impairment (MCI), and cognitively healthy controls (HC). We stratified participants by *APOE* ε4 carrier status, AAO, and sex, to assess how *CHI3L1* expression varies across genetically and clinically defined subgroups. To explore its relationship to systemic immune activation, we examined correlations between *CHI3L1* and a panel of inflammatory gene transcripts, including *IL1B*, *TNF*, *MMP9*, *LRP1*, and *TREM2.* By characterizing peripheral *CHI3L1* in AD continuum, we aimed to gain insight into systemic inflammatory gene signatures and to identify expression patterns relevant to *APOE* genotype, sex and clinical phenotype.

## Methods

### Study cohort

Peripheral blood samples were obtained by clinical staff at the Department of Neurology, Vienna General Hospital, during routine diagnostic procedures. The study cohort included individuals diagnosed with Alzheimer’s disease (AD, *n* = 34), mild cognitive impairment in the context of AD (MCI, *n* = 31), and age- and sex-matched controls without neurological disease (HC, *n* = 21). Diagnostic classifications were based on NIA-AA criteria for biological AD definition, including neuropsychological testing, magnetic resonance imaging (MRI) and amyloid biomarker assessments (Albert et al., 2011; Jack et al., 2018). Cognitive function was assessed with the Neuropsychological Test Battery Vienna (NTBV) covering attention, language, executive functions, and episodic memory, with age-, education-, and sex-corrected z-scores derived from normative data (Lehrner et al., 2015; Pusswald et al., 2013). Global cognition was evaluated by Mini-Mental State Examination (MMSE) and Wortschatztest (WST) as an estimate of premorbid IQ, and depressive symptoms were assessed using the Beck Depression Inventory-II (BDI-II) (Kühner et al., 2007).

All patients underwent at least T1-weighted, T2-weighted or fluid-attenuated inversion recovery (FLAIR), and diffusion-weighted MRI sequences as part of routine diagnostics to evaluate atrophy, vascular lesions, and to exclude alternative pathologies or diffusion-restricted areas. Amyloid-PET was performed in 29 MCI and 28 AD patients using either [^11^C] Pittsburgh compound-B (PiB, *n* = 52) or [^18^F] flutemetamol (Vizamyl®, *n* = 28) on a Siemens Biograph 64 True Point or GE Advance PET scanner. Approximately 400 MBq [^11^C] PiB or 185 MBq [^18^F] flutemetamol were administered intravenously, with image acquisition starting 40 min (PiB) or 90 min (flutemetamol) post-injection and lasting ~20 min, following CT-based attenuation correction (Philippe et al., 2011). Scans were visually rated as amyloid-positive or -negative by an experienced nuclear medicine physician according to manufacturer guidelines.

CSF was collected from 19 MCI and 18 AD patients by lumbar puncture (L3/L4, L4/L5, or L5/S1) into polypropylene tubes and stored at −20 °C until biomarker analysis (Aβ42, pTau181, tTau) or immediately at −80 °C for research purposes. Concentrations of Aβ42, pTau181, and tTau were measured using commercial ELISAs (Innotest, Fujirebio), applying manufacturer cut-offs (Aβ42 < 500 pg./mL, pTau181 > 61 pg./mL, tTau > 300 pg./mL) (Vanderstichele et al., 2000; Vanmechelen et al., 2000). From these measures, the Innotest Amyloid Tau Index (IATI = Aβ42/(240 + 1.18 × tTau)) was calculated, with values <1 indicating AD pathology (Tabaraud et al., 2012).

Only patients with either positive CSF biomarkers or positive Amyloid PET and MRI results were included in the disease study cohort. For 19 MCI patients and 15 AD patients, both PET and CSF data was available. To distinguish sporadic from monogenic early-onset AD, individuals with age at onset ≤65 years and a positive family history who consented to genetic testing underwent whole-exome sequencing (WES). In total, 12 of 15 EOMCI patients (80%) and 14 of 21 EOAD patients (67%) were genetically evaluated, and no pathogenic variants in APP, PSEN1, or PSEN2 were identified; such cases were excluded from the cohort by design.

The remaining early-onset patients who did not undergo sequencing all had a Goldman score (GS) of 3.5–4, which indicates one relative with late-onset dementia (GS 3.5) or no known family history of dementia (GS 4), both of which carry a very low likelihood of autosomal-dominant AD (Goldman et al., 2005).

Further exclusion criteria included active systemic inflammatory or autoimmune diseases. Control participants had no history of neurological disease and no comorbidities known to affect systemic inflammation.

Peripheral blood sampling for RNA extraction was performed as part of the clinical diagnostic work-up. CSF collection and/or PET imaging occurred within the same diagnostic episode, though not necessarily on the same day. No participant was receiving systemic corticosteroids or immunosuppressive therapy at the time of sampling. Detailed information on chronic medications (e.g., statins, antihypertensives, NSAIDs) was not systematically recorded.

### Ethics

The study was conducted in accordance with the Declaration of Helsinki and approved by the Ethics Committee of the Medical University of Vienna. Written informed consent, including publication of data in scientific journals and deposition in scientific databases, was obtained from all participants in the course of the inclusion in two existing registries: Dementia Registry RDA MUV (EK 1323/2018, approval date: 15.06.2018) and the BIOBANK MUV (EK 2195/2016, approval date: 17.02.2017).

### Genotyping

DNA was isolated from whole blood collected in EDTA tubes using standard procedures. The *APOE* genotype was determined in genomic DNA from patients using TaqMan qPCR with probes for two single nucleotide polymorphisms (SNPs) in the *APOE* gene, rs429358 and rs7412. Fluorescence of VIC and FAM, tagging the two different SNPs in each probe, was measured, enabling identification of ε2, ε3, and ε4 alleles.

### RNA isolation from whole-blood samples and gene expression analysis

Blood was collected in PAXgene Blood RNA tubes (Qiagen, 762,165) and stored at −20 °C until processing. RNA was extracted using the PAXgene Blood RNA Kit (PreAnalytiX, 762,164) following the manufacturer’s protocol. RNA concentration and purity were assessed via NanoDrop spectrophotometry (Thermo Fisher Scientific), ensuring 260/280 ratios between 1.9 and 2.1. Samples were stored at −80 °C prior to cDNA synthesis. cDNA was synthesized from 500 ng to 1 μg total RNA using the iScript™ cDNA Synthesis Kit (Bio-Rad, 1,708,891) according to the manufacturer’s instructions.

### Quantitative PCR

Quantitative PCR (qPCR) was performed in technical duplicates on an AriaMx Real-time PCR System (Agilent), using SYBR Green- and probe-based assays as appropriate. For SYBR Green assays, reactions were assembled with SsoAdvanced™ Universal SYBR® Green Supermix (Bio-Rad, 1725272) and gene-specific primers (Sigma-Aldrich; sequences listed in Supplementary Table 1). The inflammatory and vascular transcripts included in this study were selected as part of a predefined, literature-based mechanistic panel reflecting pathways relevant to *CHI3L1* biology. Specifically, *IL1B* and *TNF* represent canonical pro-inflammatory cytokines; *MMP9* and LRP1 as mediators of matrix remodeling, vascular integrity, and BBB permeability; and *TREM2* reflects peripheral myeloid activation pathways associated with genetic AD risk. These analyses were designed as exploratory correlations within this predefined mechanistic panel rather than an unbiased transcriptomic screen. *APOE* transcript quantification used PrimePCR™ Probe Assays (Bio-Rad) with Luna® Universal Probe qPCR Master Mix (NEB, M3003L), and *TREM2* quantification employed a predesigned TaqMan Assay (ThermoFisher, Assay ID C_100657057_10).

### Housekeeping genes, normalization, and quality control

Relative gene expression was calculated on the ΔCt scale. *GAPDH* served as the reference gene for SYBR Green-based assays, while *RNase P* was used for probe-based assays (TaqMan and PrimePCR). All statistical inference was performed on ΔCt values, with lower ΔCt corresponding to higher transcript abundance. Fold-change values were derived by normalizing each sample’s ΔCt to the median ΔCt of the healthy control group (2^-ΔΔCt) and were log₂-transformed for visualization. As this transformation can amplify variance, particularly at extreme values in small subgroups, fold-change plots are provided for graphical purposes only, whereas all statistical analyses and effect-size estimates were based on ΔCt values.

To verify the appropriateness of normalization procedures, we assessed the stability of the housekeeping genes *GAPDH* and *RNase P* across diagnostic groups, *APOE* ε4 genotypes, and age. Raw Ct values were compared using Kruskal-Wallis tests, Dunn’s post-hoc comparisons, and Spearman correlations, and coefficients of variation (CV) were calculated to quantify dispersion. Both genes showed narrow Ct distributions with low variability (*GAPDH*: mean 20.55 ± 1.88, CV = 9.1%; *RNase P*: mean 23.00 ± 1.59, CV = 6.9%), consistent with stable expression across samples. Neither *GAPDH* nor *RNase P* Ct values differed between *APOE* ε4 carriers and non-carriers (all *p* > 0.05), and Ct values did not correlate with age within HC, MCI, or AD (all *p* > 0.10). *GAPDH* Ct values were also stable across diagnostic groups, whereas *RNase P* showed a modest group-wise effect (Kruskal-Wallis *p* < 0.01), driven by a significant HC-AD difference in Dunn’s post-hoc testing (adjusted *p* = 0.0016), while HC-MCI and MCI-AD comparisons were non-significant (Supplementary Figure 2). Because *RNase P* was used exclusively for normalization of the probe-based *TREM2* assay, we performed sensitivity analyses in which *TREM2* expression was re-normalized to *GAPDH* alone and to the geometric mean of *GAPDH* and *RNase P*. Across all normalization strategies, *CHI3L1*-*TREM2* correlations remained non-significant, demonstrating that technical differences in reference scaling did not materially influence outcomes.

To assess potential batch-related or temporal drift effects, we examined associations between measurement order and Ct values of *GAPDH* and *RNase P* as well as ΔCt values of *CHI3L1*. *GAPDH* Ct values showed a weak negative association with measurement order in the AD group [*ρ* = −0.492, *p* = 0.003; 95% CI (−0.791, −0.093)], but the corresponding ΔCt values were unaffected [*ρ* = 0.038, *p* = 0.829; 95% CI (−0.362, 0.436)], indicating successful normalization. *RNase P* Ct values showed no significant temporal structure in any group, and no systematic drift was observed for *CHI3L1* ΔCt values across diagnostic categories.

RNA quality was assessed in a representative subset of samples (*n* = 5 per diagnostic group) using High Sensitivity RNA ScreenTape analysis (Agilent). All samples demonstrated integrity acceptable for qPCR analyses (mean RIN = 5.8 ± 2.0) with no group-wise differences. To evaluate potential effects of RNA quality on qPCR performance, correlations between RIN and Ct values for *GAPDH*, *RNase P*, and *CHI3L1* were examined; no significant associations were observed (all *p* > 0.05; 95% CIs spanning zero), indicating that variability in RNA integrity did not influence housekeeping gene amplification or relative quantification. Amplification efficiency (E) was determined from a 5-point dilution series and accepted if 90–110% (*R*^2^ ≥ 0.99). Primer specificity for SYBR Green assays was verified by melt curve analysis. Duplicate reactions with SD > 0.5 Ct were repeated, and no-template as well as no-reverse transcription controls were included on each plate. Samples from all diagnostic groups (HC, MCI, and AD) were distributed across plates to avoid group-plate confounding. All plates were processed under identical cycling conditions, reactions were run in technical duplicates, and ΔCt normalization minimized plate-to-plate variation. Amplification efficiencies were consistent across plates, confirming robust assay performance.

### Statistical analysis

Statistical analyses were conducted using GraphPad Prism 8 (GraphPad Software, San Diego, CA).

*Analysis hierarchy and exploratory framework*: To align analytic complexity with the limited cohort size (*n* = 86), analyses were structured according to a predefined hierarchy.

*Primary analysis*: Group-wise comparison of *CHI3L1* ΔCt stratified by *APOE* ε4 carrier status and AAO (Mann Whitney U; corrected with Holm-Šidák).

*Secondary analyses*:

(a) Correlations between *CHI3L1* and a predefined, literature-based panel of mechanistically relevant inflammatory transcripts (*IL1B, TNF, MMP9, LRP1, TREM2*), selected for their roles in immune activation, extracellular matrix remodeling, and blood–brain barrier function [Spearman correlation; corrected with Benjamini-Hochberg false discovery rate (FDR)].
(b) Post-hoc stratifications by sex and AAO, performed to generate hypotheses for future studies (Spearman correlation; corrected with Benjamini-Hochberg FDR).

All stratified and correlation results are interpreted as exploratory, with emphasis on effect sizes and confidence intervals rather than statistical significance.

Data normality was assessed using the Shapiro–Wilk test. Given the non-normal distribution of gene expression data (ΔCt values), non-parametric tests were applied for group comparisons and correlations. All analyses were performed on ΔCt values, which represent relative cycle differences.

#### Power analysis

To evaluate the sensitivity of our EOAD subgroup comparison (*APOE* ε4 carriers vs. non-carriers), we performed an a-priori power analysis with unequal sample allocation reflecting the expected *APOE* ε4 carrier ratio in EOAD (ε4+:ε4 − ≈ 1.6). Power was computed using pwr.t2n.test (two-sided *α* = 0.05) and validated via Monte Carlo simulations of the Wilcoxon test. Assuming conservative-to-intermediate effect sizes (Cohen’s d = 0.6–0.9), achieving 80–90% power would require total sample sizes of approximately *N* = 45–126. Given our EOAD subgroup (*n* = 21; 13 ε4+, 8 ε4−), the study had sensitivity for large effects; smaller effects were underpowered. Post-hoc analyses were conducted to estimate the statistical power of the current dataset. Mean ΔCt values showed only moderate group differences and pairwise comparisons yielded small-to-medium effect sizes (Cohen’s d = 0.19–0.67), corresponding to estimated powers of approximately 0.1–0.8 (*α* = 0.05). The overall omnibus group comparison (Kruskal-Wallis) power was ~0.44, indicating that the study is underpowered for detecting subtle group differences. Findings should therefore be interpreted as exploratory and hypothesis generating, rather than confirmatory.

#### Group comparisons

Multi-group comparisons of *CHI3L1* expression across diagnostic categories (HC, MCI, AD) and AAO subgroups [early-onset MCI (EOMCI), late-onset MCI (LOMCI), early-onset AD (EOAD), late-onset AD (LOAD)] were conducted using Kruskal-Wallis tests, followed by Dunn’s *post hoc* procedure. Binary comparisons between *APOE* ε4 carriers and non-carriers within diagnostic or onset subgroups were performed using Mann–Whitney U tests. Hodges-Lehmann estimator of the median difference (ΔCt) with the exact confidence interval derived from the Mann–Whitney distribution, alongside the exact two-tailed *p*-value are reported. Where multiple pairwise comparisons were conducted, Holm-Šidák correction was applied, and corrected *p*-values are reported. For subgroup comparisons with small sample sizes in our primary analysis, the robustness of location estimates was assessed using non-parametric bootstrapping (10,000 iterations) Hodges-Lehmann median shifts and Hedges’ g were recalculated and 95% percentile-based confidence intervals were derived.

#### Regression analyses

To account for potential demographic and diagnostic confounders, multivariable linear regression models were fitted with *CHI3L1* ΔCt as the dependent variable. Predictors included age at blood draw, sex, *APOE* ε4 carrier status, and diagnostic group, with interaction terms (e.g., *APOE* × cohort or *APOE* × onset category) where appropriate. Separate models were run for EOAD and LOAD to assess genotype-specific effects within onset categories. Chronological age was included as a demographic covariate, while AAO was used for subgroup stratification given its relevance to biological subtype definition. Disease duration was not modeled to avoid collinearity with age and AAO in this exploratory cohort. Regression model *p*-values are adjusted for covariates and corrected with Benjamini-Hochberg FDR.

#### Correlation analyses

Associations between *CHI3L1* expression and inflammatory gene transcripts (*IL1B*, *TNF*, *TREM2*, *LRP1*, and *MMP9*) were assessed using Spearman’s rank correlation within diagnostic and onset subgroups. *APOE* ε4-stratified analyses were pre-specified, given our genotype-dependent hypotheses, whereas sex-stratified analyses were exploratory follow-up analyses conducted to examine potential modulation of genotype effects. Multiple testing correction was applied to correlation analyses using the Benjamini-Hochberg FDR procedure (two-sided; q reported). Statistical significance was set at q < 0.05 (two-tailed). In additional exploratory analyses, we examined associations of *CHI3L1* with systemic inflammatory and lipid markers (CRP, total cholesterol, HDL, LDL) using Spearman’s correlation within diagnostic and onset subgroups. Unadjusted *p*-values and adjusted q-values, along with model coefficients (r), are provided in Supplementary Tables 3, 4.

#### Multiplicity control

Family-wise error for pairwise group tests was controlled with Holm-Šidák (primary analysis). For regression families (global model + stratified EOAD/LOAD models considered *a priori*) and for all correlation panels (including sex- and *APOE*-stratified exploratories), *p*-values were adjusted using Benjamini-Hochberg FDR within each analysis family (secondary analyses); we report q where applicable.

## Results

### Peripheral *CHI3L1* expression is selectively increased in early-onset Alzheimer’s disease

The final cohort consisted of 31 biomarker-confirmed MCI patients, 34 biomarker-confirmed AD patients, and 21 age- and sex-matched HC (Table 1). For consistency, all statistical tests were based on ΔCt values, with lower ΔCt reflecting higher expression. Fold-change values shown in figures reflect only visual scaling and were not used for hypothesis testing. To evaluate potential demographic influences, we first assessed the relationship between *CHI3L1* ΔCt values, age at blood draw, and sex. In unadjusted analyses, no significant sex differences were observed within any diagnostic group, and bivariate correlations with age were weak (Supplementary Figure 1). However, when considered in a multivariable regression model including diagnostic group, *APOE* status, age, and sex, age emerged as a significant predictor of *CHI3L1* ΔCt (*β* = +0.064, *p* = 0.004, *q* = 0.020), indicating lower *CHI3L1* expression with increasing age. Sex, *APOE* ε4 status, and diagnostic cohort were not significant independent predictors (all *p* > 0.44). Likewise, the *APOE* ε4 × cohort interaction showed no association (*β* = −1.058, *p* = 0.060, *q* = 0.150) (see Supplementary Table 2A).

**Table 1:** Characterization of the study cohort by diagnosis, sex, AAO, age at blood draw, MMSE score and *APOE* status.

Characteristics	HC	MCI			AD		
	Total	EO	LO	Total	EO	LO	Total
Individuals (*n*)	21	15	16	31	21	13	34
Sex (% female)	57.1	53.3	68.8	61.3	66.7	53.8	61.8
Median age (range)	61 (51–82)	60 (43–74)	74 (68–79)	70 (43–79)	59 (49–74)	75 (67–79)	66 (49–79)
Median AAO (range)	n.a.	57 (40–64)	70 (66–79)	67 (40–79)	57 (46–65)	71 (66–78)	64 (46–78)
Median MMSE	n.a.	27.0	26.0	26.5	20.0	21.0	20.0
*APOE* 4 + (*n*, %)	5 (23.8)	6 (40.0)	11 (68.8)	17 (54.8)	13 (61.9)	10 (76.9)	23 (67.6)
*APOE* 4/4 (*n*, %)	0 (0)	4 (26.7)	3 (18.8)	7 (22.6)	3 (14.3)	3 (23.1)	5 (14.7)

HC, healthy controls; MCI, mild cognitive impairment; AD, Alzheimer’s disease; AAO, age at onset; EO, early-onset; LO, late-onset; MMSE, mini-mental state examination; APOE, apolipoprotein E; APOE 4+, hetero- and homozygote carriers.

**Figure 1:**
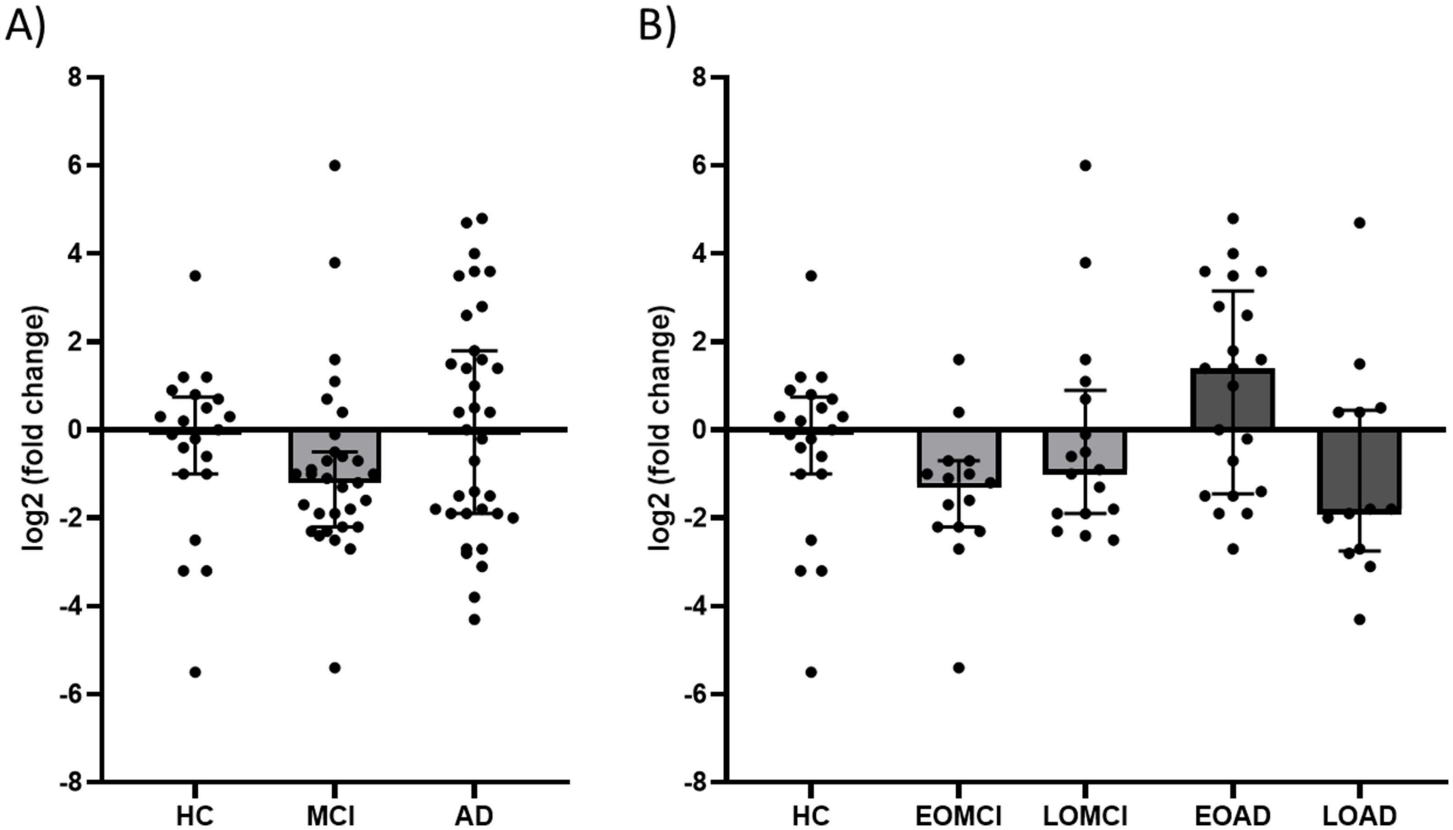
CHI3L1 Expression across cohorts and stratified by AAO. **(A)** *Log_2_* fold change of ΔCt *CHI3L1* values in HC, MCI, and AD. **(B)** *Log_2_* fold change of *CHI3L1* ΔCt values in HC, EOMCI, LOMCI, EOAD, and LOAD. Data are presented as individual data points with bars indicating the median and interquartile range. The horizontal line at zero represents the expression level of healthy controls. Statistical tests were performed on ΔCt values; log₂ fold-change values are shown for visualization only. HC, healthy controls; MCI, mild cognitive impairment; AD, Alzheimer’s disease; EO, early-onset; LO, late-onset; *CHI3L1*, chitinase-3-like protein 1.

Comparison of *CHI3L1* transcript levels across HC, MCI, and AD groups revealed no significant differences (*p* = 0.174). Higher median *CHI3L1* ΔCt values (indicating lower expression) within the MCI group relative to HC and AD did not reach significance (HC vs. MCI, *p* = 0.486 and MCI vs. AD, *p* = 0.241 after Dunn’s correction; Figure 1A).

To further investigate whether age at onset (AAO) modulates peripheral *CHI3L1* expression, we stratified the MCI and AD cohorts into early-onset (EO; AAO < 65 years), and late-onset (LO; AAO ≥ 65 years) subgroups. Kruskal-Wallis testing revealed a significant overall group effect when comparing HC (*n* = 21), EOMCI (*n* = 15), LOMCI (*n* = 16), EOAD (*n* = 21), and LOAD (*n* = 13) groups (*H* = 12.02, *p* = 0.017), with an effect size of *η*^2^ = 0.10 (95% CI 0.00 to 0.13). This effect was primarily attributable to increased *CHI3L1* expression in the EOAD subgroup, which exhibited the lowest ΔCt values (indicating highest expression levels) across all groups (Figure 1B). Although *post hoc* pairwise comparisons did not reach significance after multiple testing correction, the expression pattern suggests selective upregulation of peripheral *CHI3L1* in EOAD. Given the modest cohort size, these subgroup patterns are interpreted as exploratory and descriptive rather than confirmatory.

### Genotype-specific upregulation of *CHI3L1* in early-onset Alzheimer’s disease is limited to *APOE* ε4 carriers

**Figure 2:**
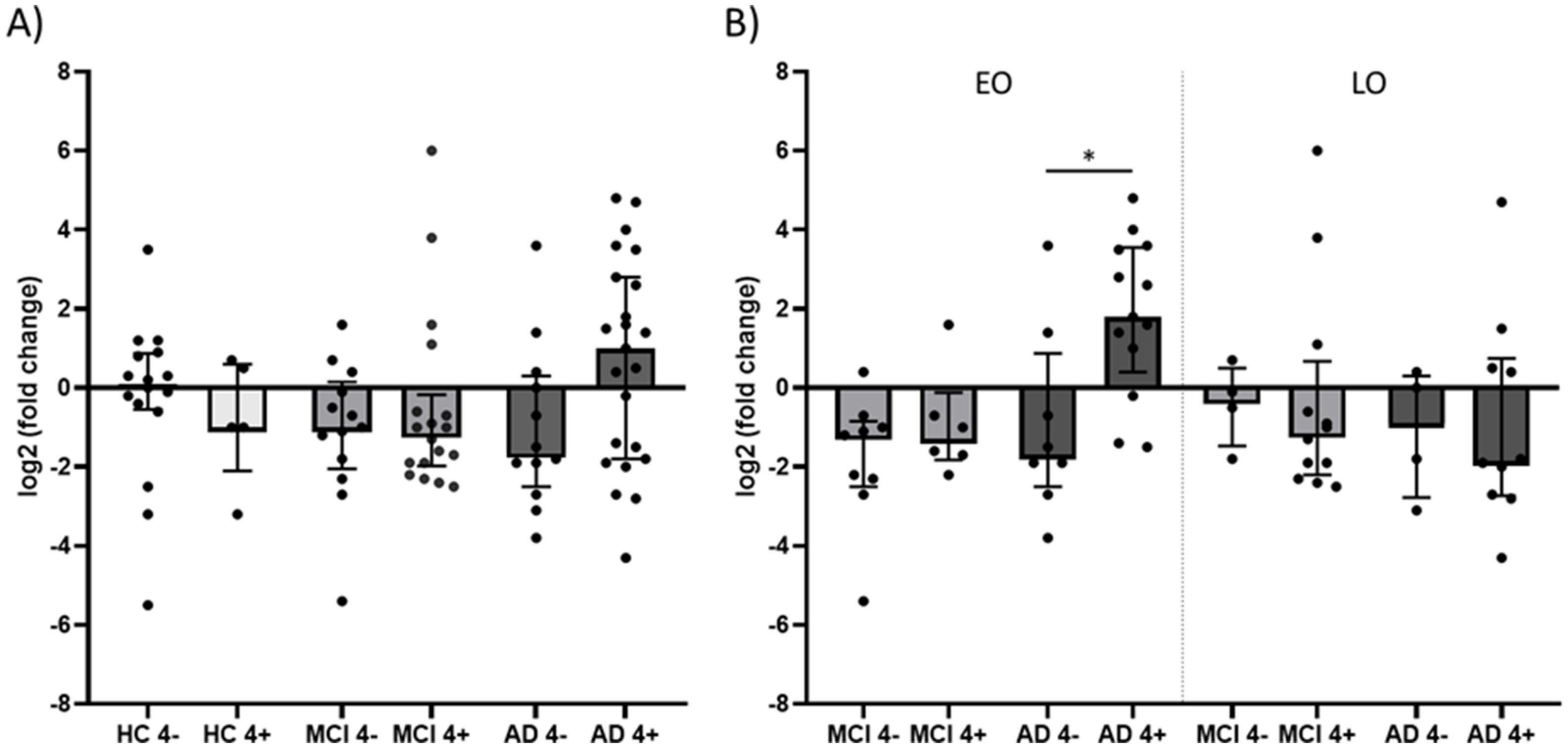
Peripheral CHI3L1 expression in HC, MCI, and AD stratified by APOE ε4 carrier status and age at onset. **(A)** Log₂ fold change of *CHI3L1* expression in HC, MCI, and AD participants, stratified by *APOE* ε4 carrier status (ε4 + vs. ε4−). **(B)** Log₂ fold change of *CHI3L1* expression in MCI and AD participants, further stratified by AAO (EO < 65 years; LO ≥ 65 years) and *APOE* ε4 status. **(A,B)** Fold changes were calculated using the ΔΔCt method relative to the median ΔCt of the pooled HC group and are displayed as log₂-transformed values. Data are shown as individual data points with bars representing the median and interquartile range. The line at zero indicates the expression level of HC. Statistical comparisons displayed in the figure were performed with ΔCt values using Mann–Whitney U tests between *APOE* ε4 + and *APOE* ε4 − individuals within each subgroup. *p*-values were corrected for multiple testing using Holm-Šidák method. To account for potential demographic and diagnostic confounders, corresponding multivariable regression analyses were additionally performed (see Results and Supplementary Table 2). *p*-values <0.05 were considered statistically significant. HC, healthy controls; MCI, mild cognitive impairment; AD, Alzheimer’s disease; *APOE* ε4+, *APOE* ε4 carriers; *APOE* ε4−, *APOE* ε4 non-carriers; EO, early-onset; LO, late-onset; AAO, age at onset; *CHI3L1*, chitinase-3-like protein 1.

We subsequently investigated whether *APOE* ε4 carrier status modulates peripheral *CHI3L1* expression. All statistical tests are based on ΔCt values, with lower ΔCt reflecting higher expression. Fold-change values shown in figures reflect only visual scaling and were not used for hypothesis testing. In HC and MCI groups, *CHI3L1* expression did not differ between *APOE* ε4 + (HC, *n* = 5; MCI, *n* = 18) and *APOE* ε4- (HC, *n* = 16; MCI, *n* = 13), indicating no genotype effect during prodromal dementia stages. While median *CHI3L1* ΔCt values (indicating higher expression) within the AD group were lower in *APOE* ε4 carriers (*n* = 23; median = 0.60, IQR: −1.20 to 3.4) compared to non-ε4 carriers (*n* = 12; median = 3.25, IQR: 1.30 to 4.10), significance was lost after Holm-Šidák correction (*p* = 0.061; Hodges-Lehmann median difference = −1.7; 95% CI = −3.6 to 0; Figure 2A). Because the global multivariable regression (Supplementary Table 2A) indicated that *APOE* ε4 effects may differ across diagnostic groups, we performed stratified regression analyses in EOAD and LOAD. In EOAD, *APOE* ε4 carrier status was the only significant predictor of *CHI3L1* expression (*β* = −2.155, 95% CI -4.019 to −0.291, *p* = 0.025), however, significance was lost after FDR correction for multiple testing (*q* = 0.075). In contrast, no predictors - including *APOE* ε4 - were significant in the LOAD subgroup (all *p* > 0.30; Supplementary Table 2B).

To investigate the interaction between *APOE* ε4 genotype and AAO, we stratified the MCI and AD cohorts into EO and LO subgroups. Mann–Whitney U tests with Holm-Šidák correction revealed no significant differences in *CHI3L1* expression between *APOE* ε4 + and *APOE* ε4- carriers in EOMCI (ε4+: *n* = 6, median = 2.90, IQR: 1.73 to 3.43; ε4-: *n* = 9, median = 2.80, IQR: 2.45 to 4.10), LOMCI (ε4+: *n* = 12, median = 2.75, IQR: 0.93 to 3.80; ε4-: *n* = 4, median = 1.90, IQR: 1.10 to 3.08), or LOAD (ε4+: *n* = 9, median = 3.45, IQR: 0.85 to 4.33; ε4-: *n* = 4, median = 2.50, IQR: 1.30 to 4.38) subgroups (all *p* > 0.999).

Notably, in the EOAD subgroup, *APOE* ε4 *+* showed significantly lower ΔCt values (higher expression) of *CHI3L1* expression compared to their non-carrier counterparts (ε4+: *n* = 13, median = −0.20, IQR: −1.95 to 1.20; ε4-: *n* = 8, median = 3.30, IQR: 0.73 to 4.10; *p* = 0.026; Hodges-Lehmann median difference = −3.3; 95.54% CI -5.3 to −0.4; Figure 2B). To assess robustness given the small subgroup sizes, we performed non-parametric bootstrap resampling (10,000 replicates) to derive confidence intervals for both the Hodges-Lehmann shift and Hedges’ g. The bootstrap-based Hodges-Lehmann estimate (−2.6; 95% CI -4.5 to −0.4) showed a comparable magnitude and direction, confirming the stability of the median difference across computational methods. The corresponding standardized effect size was large (Hedges’ g = −1.09; 95% bootstrap CI -2.61 to −0.26). Collectively, these results support the robustness of the observed genotype effect despite limited subgroup size.

### *CHI3L1* expression reflects markers of systemic inflammation

We examined associations between *CHI3L1* and transcripts from a predefined, literature-based mechanistic panel of disease-related genes as hypothesis-driven exploratory analyses to characterize peripheral inflammatory pathways potentially linked to *CHI3L1* expression. For this we performed Spearman correlation analyses and FDR was controlled using the Benjamini-Hochberg procedure.

Genes of interest included classical inflammatory mediators (*IL1B, TNF, TREM2*) and BBB-associated factors (*LRP1, MMP9*). Spearman correlations were performed on ΔCt values, with lower ΔCt reflecting higher expression.

In the HC group, no significant correlations were observed between *CHI3L1* expression and any of the examined markers. In the MCI cohort, *CHI3L1* expression correlated positively with *MMP9* (*r* = 0.631, *q* < 0.001; CI 0.33 to 0.82). In AD, *CHI3L1* exhibited positive correlations with *MMP9* (*r* = 0.663; *q* < 0.0001; CI 0.41 to 0.82), *IL1B* (*r* = 0.480; *q* = 0.007; CI 0.17 to 0.71), *TNF* (*r* = 0.395; *q* = 0.024; CI 0.06 to 0.65) and *LRP1* (*r* = 0.745; *q* < 0.0001; CI 0.54 to 0.87).

Stratification by AAO revealed no significant correlations in EOMCI. In LOMCI, *CHI3L1* correlated with *TNF* (*r* = 0.690; *q* = 0.008; CI 0.29 to 0.89) and *MMP9* (*r* = 0.780; *q* = 0.008; CI 0.39 to 0.93). Within the AD group, correlations were observed in EOAD with *TNF* (*r* = 0.480; *q* = 0.040; CI 0.06 to 0.76) *LRP1* (*r* = 0.785; *q* < 0.0001; CI 0.53 to 0.91) and *MMP9* (*r* = 0.805; *q* < 0.0001; CI 0.56 to 0.92), but not in LOAD (Table 2).

**Table 2:** Overview of correlation of CHI3L1 with immunological and inflammatory markers stratified by AAO.

Measure	*IL1B*	*TNF*	*LRP1*	*TREM2*	*MMP9*
HC					
Spearman r	0.017	0.051	0.246	0.060	0.427
*p* (two-tailed)	0.941	0.825	0.283	0.798	0.088
*q* value	0.941	0.941	0.708	0.941	0.440
Significance	ns	ns	ns	ns	ns
MCI					
Spearman r	0.163	0.251	0.397	−0.169	0.631
*p* (two-tailed)	0.382	0.167	0.024	0.357	<0.001
*q* value	0.382	0.278	0.060	0.382	**<0.001**
Significance	ns	ns	ns	ns	**
EOMCI					
Spearman r	0.038	−0.261	0.351	0.056	0.466
*p* (two-tailed)	0.894	0.344	0.199	0.843	0.081
*q* value	0.894	0.573	0.498	0.894	0.405
Significance	ns	ns	ns	ns	ns
LOMCI					
Spearman r	0.279	0.690	0.495	−0.414	0.780
*p* (two-tailed)	0.293	0.003	0.045	0.099	0.002
*q* value	0.293	**0.008**	0.075	0.124	**0.008**
Significance	ns	**	ns	ns	**
AD					
Spearman r	0.480	0.395	0.745	0.288	0.663
*p* (two-tailed)	0.004	0.019	<0.00001	0.105	<0.0001
*q* value	**0.007**	**0.024**	**<0.0001**	0.105	**<0.0001**
Significance	**	*	****	ns	****
EOAD					
Spearman r	0.315	0.480	0.785	0.262	0.805
*p* (two-tailed)	0.153	0.024	<0.0001	0.252	<0.0001
*q* value	0.191	**0.040**	**<0.0001**	0.252	**<0.0001**
Significance	ns	*	****	ns	****
LOAD					
Spearman r	0.663	0.347	0.503	0.305	0.505
*p* (two-tailed)	0.016	2.438	0.812	3.321	0.963
*q* value	0.081	0.308	0.162	0.335	0.162
Significance	ns	ns	ns	ns	ns

HC, healthy controls; MCI, mild cognitive impairment; AD, Alzheimer’s disease; AAO, age at onset; EO, early-onset; LO, late-onset; CHI3L1, chitinase-3-like protein; IL1B, interleukin-1 beta; TNF, tumor necrosis factor; LRP1, low-density lipoprotein receptor-related protein 1; TREM2, triggering receptor expressed on myeloid cells 2; MMP9, matrix metalloproteinase 9. Bold values indicate significant findings.

**Figure 3:**
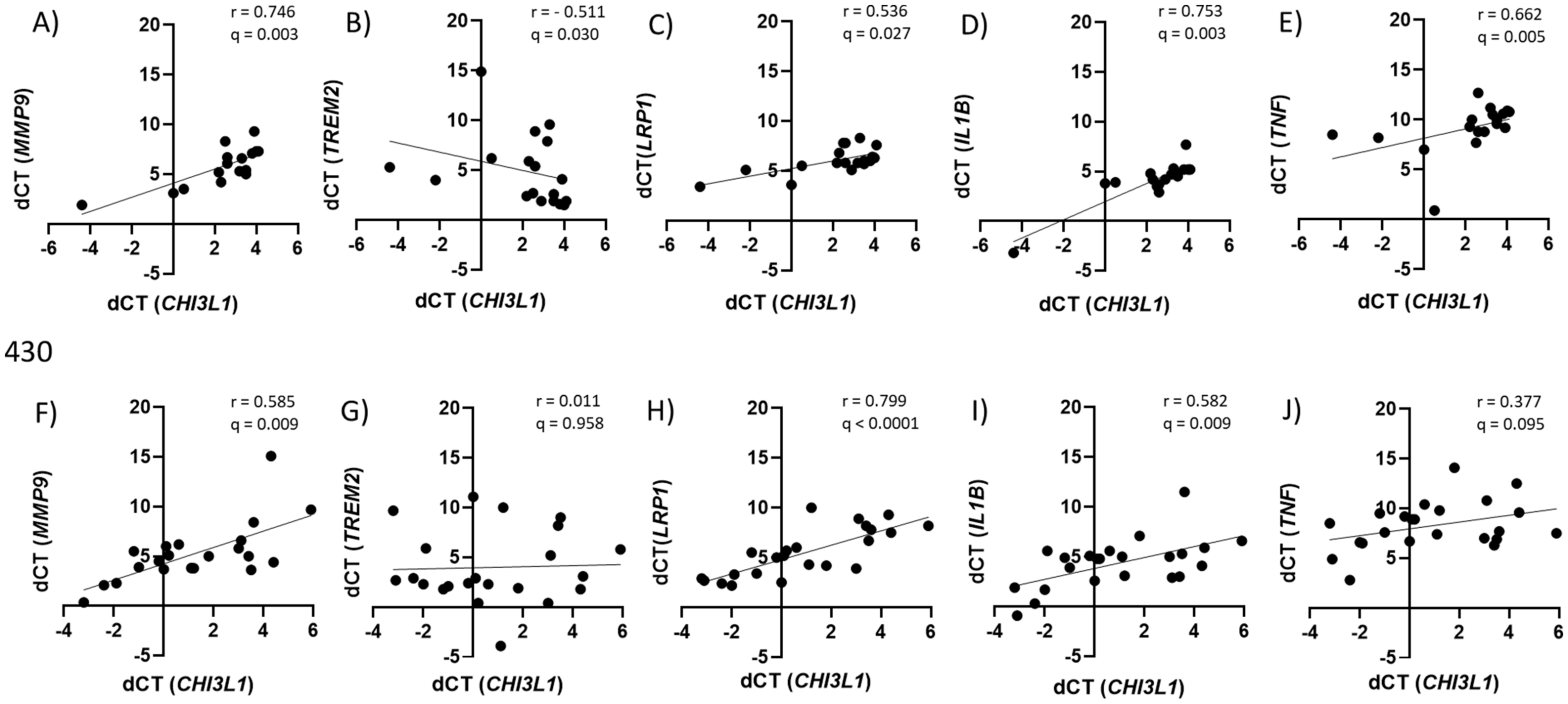
Correlations between peripheral CHI3L1 expression and inflammatory markers in APOE ε4 + MCI and AD patients. **(A–E)** Correlations between *CHI3L1* ΔCt values and inflammatory markers (*IL1B*, *TNF*, *LRP1*, *TREM2*, *MMP9*) in *APOE* ε4 *+* MCI. **(F–J)** Correlations between *CHI3L1* ΔCt values and inflammatory markers (*IL1B*, *TNF*, *LRP1*, *TREM2*, *MMP9*) in *APOE* ε4 *+* AD. Lower ΔCt values indicate higher *CHI3L1* expression. Spearman correlation coefficients (r) and q-values are shown (FDR adjusted); q-values < 0.05 were considered statistically significant. MCI, mild cognitive impairment; AD, Alzheimer’s disease; *APOE* ε4*+*, *APOE* ε4 carriers; *IL1B*, interleukin-1 beta; *TNF*, tumor necrosis factor; *LRP1*, low-density lipoprotein receptor-related protein 1; *TREM2*, triggering receptor expressed on myeloid cells 2; *MMP9*, matrix metalloproteinase 9; *CHI3L1*, chitinase-3-like protein.

When stratifying by *APOE* ε4 status, no significant correlations were identified in MCI *APOE* ε4 *−* individuals. In contrast, MCI *APOE* ε4 *+* participants showed correlations between *CHI3L1* and *IL1B* (*r* = 0.753; *q* = 0.003; CI 0.41 to 0.91), *TNF* (*r* = 0.662; *q* = 0.005; CI 0.27 to 0.87), *LRP1* (*r* = 0.536; *q* = 0.027; CI 0.08 to 0.81), *TREM2* (*r* = −0.511; *q* = 0.030; CI -0.79 to −0.04), and *MMP9* (*r* = 0.746; *q* = 0.003; CI 0.38 to 0.91) (Figures 3A–E). In the AD cohort, no significant associations were found in *APOE* ε4 *−* individuals. In *APOE* ε4 *+* AD individuals, *CHI3L1* correlated with *IL1B* (*r* = 0.582; *q* = 0.009; CI 0.21 to 0.81), *LRP1* (*r* = 0.799; *q* < 0.0001; CI 0.57 to 0.91), and *MMP9* (*r* = 0.585; *q* = 0.009; 0.19 to 0.82) (Figures 3F–J).

Notably, exploratory sex-stratified analyses revealed that the association between *CHI3L1* expression and inflammatory markers was strongest in female *APOE* ε4 + individuals. In this subgroup, *CHI3L1* expression correlated significantly with *MMP9* (*r* = 0.788; *q* = 0.010; CI 0.37 to 0.94), *IL1B* (*r* = 0.705; *q* = 0.015; CI 0.24 to 0.91), *TNF* (*r* = 0.757; *q* = 0.010; CI 0.34 to 0.93), and *TREM2* (*r* = −0.646; *q* = 0.025; CI -0.89 to −0.13) in MCI (Supplementary Figures 3A–E) and with *IL1B* (*r* = 0.726; *q* = 0.011; CI 0.30 to 0.91), *LRP1* (*r* = 0.833; *q* = 0.002; CI 0.53 to 0.95), and *MMP9* (*r* = 0.706; *q* = 0.022; CI 0.20 to 0.91) in AD (Supplementary Figures 3F–J). These correlations were not observed in males or in *APOE* ε4- females, suggesting a potential sex-by-genotype interaction in peripheral immune activation (Supplementary Table 3).

Exploratory associations with CRP and lipids showed that *CHI3L1* expression did not correlate with CRP or lipid markers in healthy controls or MCI (all *p* > 0.05), arguing against baseline systemic inflammation or dyslipidemia as primary drivers of group differences. In AD, modest inverse correlations were observed with total cholesterol (Spearman *r* = −0.476, *q* = 0.032; CI -0.74 to −0.09), as well as HDL (*r* = −0.444, *q* = 0.045; CI -0.73 to −0.03) and LDL (*r* = −0.532, *q* = 0.032; CI -0.78 to −0.14), whereas no significant associations were detected in EOAD or LOAD when analyzed separately. These data suggest limited confounding by subclinical inflammation in controls and prodromal disease, with evidence for an inflammatory-metabolic interplay in established AD (Supplementary Table 4).

## Discussion

In this study, we investigated peripheral *CHI3L1* expression across the Alzheimer’s disease (AD) spectrum, stratified by *APOE* genotype, age at onset (AAO), and sex. We identified a selective increase in *CHI3L1* transcript levels in early-onset AD (EOAD), particularly among *APOE* ε4 carriers. *CHI3L1* expression also correlated with peripheral inflammatory markers, with the strongest associations observed in *APOE* ε4 + individuals and evidence of sex-specific effects. Because measurements were derived from whole blood, peripheral *CHI3L1* transcript levels should be interpreted as indicators of overall peripheral immune activity rather than direct measures of cell-intrinsic transcriptional regulation. Thus, elevated *CHI3L1* may reflect either increased transcript abundance within circulating myeloid cells or altered leukocyte composition in genetically susceptible EOAD patients. Importantly, subgroup sample sizes were small, these findings should be regarded as hypothesis-generating, consistent with the predefined analytical hierarchy. Exploratory correlations with CRP and circulating lipids did not indicate substantial metabolic inflammation effects in controls or mild cognitive impairment (MCI), suggesting that the EOAD/ε4-linked *CHI3L1* signal is not driven by general systemic inflammation. However, while these correlations argued against overt systemic inflammation, residual confounding by unmeasured factors such as body-mass index (BMI), metabolic or cardiovascular comorbidities cannot be excluded and should be addressed in future studies.

Peripheral *CHI3L1* expression showed a distinct pattern across diagnostic stages, with the most pronounced elevations observed in early-onset cases and particularly among *APOE* ε4 carriers. While no previous studies have directly examined peripheral *CHI3L1* mRNA in relation to AAO and *APOE* genotype, our findings align with protein-based evidence. A recent meta-analysis reported significantly elevated plasma YKL-40, the protein product of *CHI3L1*, in AD compared to HC (Zhang et al., 2023). Systematic reviews have also indicated that elevated CSF YKL-40/Aβ42 ratios and plasma YKL-40 levels are associated with brain atrophy, cognitive decline, and dementia risk, though results remain heterogeneous (Heneka et al., 2025). Choi et al. (2011) further observed elevated plasma YKL-40 in early AD but not in LOAD or MCI, consistent with our EOAD-specific increase. These parallels support the view that *CHI3L1* reflects inflammatory dynamics that are stronger in EO disease forms, potentially linked to genetic and microglial signatures previously associated with EOAD (Sun et al., 2023; Tondo et al., 2020). Of note, although EOMCI and EOAD patients carrying autosomal-dominant mutations were excluded from the study, rare monogenic cases in genetically undisclosed early-onset patients with weak or absent family history cannot be fully ruled out. Although autosomal-dominant mutations can influence inflammatory profiles in their carriers, their expected frequency among individuals with low Goldman scores (GS 3.5–4) is estimated at only approximately 5–10% within EOAD cohorts and is therefore unlikely to materially affect group-level results (Koriath et al., 2020).

While protein biomarkers are widely used in clinical research and increasingly integrated into diagnostic workflows, peripheral mRNA quantification remains a research tool. Transcript-level measurements provide a mechanistic context by capturing early peripheral immune activation that may precede detectable protein changes. Thus, although qPCR-based mRNA detection is not intended to replace immunoassays such as ELISA, transcript-level measures offer insight into whether peripheral YKL-40 elevations reflect changes in immune cell activity or abundance.

Reports on the influence of *APOE* ε4 on YKL-40 protein levels have been inconsistent. Several studies found no differences in CSF YKL-40 between carriers and non-carriers (Tondo et al., 2020; Koriath et al., 2020; Antonell et al., 2014), whereas Gispert et al. (2017) demonstrated elevated CSF YKL-40 in ε4 carriers and ε4-specific associations with brain structure. Our findings extend this literature by showing genotype-specific *CHI3L1* upregulation at the transcript level in EOAD, supporting convergence between EOAD pathology, *APOE* ε4-modulated inflammatory activity, and peripheral immune signatures.

Multivariable regression analyses clarified the contribution of demographic and diagnostic factors. Age emerged as a significant predictor of *CHI3L1* ΔCt, with higher chronological age associated with lower *CHI3L1* expression. This inverse age relationship contrasts with the elevated *CHI3L1* levels observed in younger EOAD ε4 carriers, suggesting that distinct age-linked and genotype-linked influences may coexist. These potential processes - immune aging and ε4-related early inflammatory activity - cannot, however, be statistically disentangled within the present cohort because of collinearity between age, AAO, and disease stage. Accordingly, this interpretation should be viewed as a hypothesis-level explanation pending confirmation in larger datasets. Sex, cohort, and *APOE* ε4 main effects were not significant in the combined model, whereas the *APOE* ε4 × cohort interaction showed a trend-level association, indicating potential stage-specific modulation. Stratified analyses confirmed that *APOE* ε4 was a significant predictor only in EOAD and not in LOAD, further supporting an EOAD-restricted genotype effect. Together, these analyses support a potential link between *CHI3L1* upregulation and *APOE* ε4 in EOAD, but also highlight the need for larger, adequately powered cohorts to confirm these exploratory findings.

Interestingly, *CHI3L1* expression was not elevated in EOMCI despite its clinical proximity to EOAD. This may suggest that upregulation occurs during the transition from prodromal to symptomatic phases, reflecting cumulative immune activation or disease burden. Alternatively, the absence of significance in EOMCI may be due to heterogeneity or limited statistical power. Together, these findings highlight the importance of simultaneously considering both disease stage and genetic background when interpreting peripheral immune markers in AD.

Of note, the present analyses were designed to examine disease stage-related differences rather than disease duration or temporal progression. Accordingly, age at blood draw was used as a demographic covariate to control for chronological effects, while disease duration was not modeled, both to minimize collinearity with age and AAO and because longitudinal relationships were beyond the scope of this cross-sectional dataset. Future longitudinal studies incorporating repeated measures will be required to determine how peripheral *CHI3L1* expression evolves over the disease course.

Across diagnostic stages, *CHI3L1* expression correlated with transcripts from the predefined mechanistic panel of genes linked to peripheral inflammation and extracellular matrix remodeling, including *IL1B*, *TNF*, *MMP9*, and *LRP1* - markers selected for their established role in AD-relevant immune and vascular pathways. *IL1B* and *TNF* are a central pro-inflammatory cytokines; *MMP9* contributes to extracellular matrix remodeling and blood–brain barrier (BBB) permeability, and *LRP1* regulates Aβ clearance and vascular homeostasis. In our dataset, associations were strongest in AD - particularly EOAD - suggesting a more pronounced genetically driven inflammatory phenotype. The link between *CHI3L1* and *MMP9* is notable, given its role in BBB dysfunction and interaction with *LRP1*-dependent pathways (Sampedro et al., 2015; Gispert et al., 2017; Montagne et al., 2017; Shackleton et al., 2019). Moreover, *CHI3L1* has been reported to be involved in *IL1B*/*TNF*/*MMP9* signaling cascades (Nee et al., 2004; Hu et al., 2024). These patterns may indicate that peripheral *CHI3L1* reflects inflammatory processes relevant to cerebrovascular vulnerability, although the data do not imply that peripheral YKL-40 crosses the BBB or directly affects BBB integrity. Instead, elevated peripheral *CHI3L1* likely mirrors systemic immune activation that parallels CNS processes, potentially arising from increased representation of *CHI3L1*-expressing myeloid cells in circulation rather than cell-intrinsic transcriptional upregulation. The absence of consistent correlation with *TREM2* supports pathway selectivity, indicating that *CHI3L1* covaries more strongly with vascular-inflammatory than with myeloid-activation signatures.

Stratification by *APOE* genotype revealed that these inflammatory associations were largely confined to ε4 carriers. In ε4-positive individuals with MCI, *CHI3L1* expression correlated with multiple measured inflammatory transcripts - including *IL1B*, *TNF*, *LRP1*, *TREM2*, and *MMP9* - suggesting that ε4-linked immune dysregulation may emerge early in the disease course. A similar pattern was observed in ε4-positive AD patients. These genotype-dependent associations align with evidence that *APOE* ε4 amplifies inflammatory responses and may shape peripheral immune phenotypes in AD. Nonetheless, *APOE* ε4 influences multiple physiological systems beyond inflammation, including lipid metabolism, vascular function, and stress signaling. Accordingly, the observed *CHI3L1*-*APOE* ε4 association may partly reflect these broader metabolic and vascular effects rather than a direct causal link between *APOE* genotype and immune gene expression.

Sex-stratified exploratory analyses suggested the strongest *CHI3L1*-inflammation correlations in female ε4 carriers. These results must be interpreted cautiously due to small subgroup sizes and lack of pre-specified interaction testing, potentially resulting in false-positive findings. While this qualitative pattern echoes prior evidence for heightened inflammatory vulnerability among female ε4 carriers, (Van Gool et al., 2019; Nee et al., 2004; Hu et al., 2024) the present data cannot determine whether these differences reflect true biological interactions or sampling variability. Importantly, larger, genotype-balanced cohorts will be required to test these interactions formally and these sex-related trends should be regarded as hypothesis-generating signals.

Several limitations warrant consideration. Despite application of a predefined analytical hierarchy, subgroup stratification by AAO, *APOE* genotype, and sex resulted in small analytical cells, limiting statistical power and increasing uncertainty in effect-size estimates; all stratified findings should therefore be considered exploratory. Although Holm-Šidák correction was applied to pairwise tests and FDR control in regression- and correlation analyses, residual multiplicity inherent to layered subgroup analyses remains a limitation.

While individuals with active inflammatory and autoimmune conditions were excluded, comorbid cardiometabolic conditions and chronic medications (e.g., statins, NSAIDs) were not systematically ascertained. Although these medications are generally associated with anti-inflammatory or lipid-lowering effects, their impact may vary across pathways and cell types, and differences in prescription patterns between diagnostic or genotype groups could introduce confounding by indication. Consequently, unmeasured medication effects cannot be excluded as potential contributors to peripheral *CHI3L1* variability. Exploratory analyses incorporating CRP and lipid measures argued against major confounding in controls and MCI, however, unrecorded variables such as body-mass index, metabolic syndrome, diabetes, or hypertension could have contributed to variability in peripheral *CHI3L1* expression.

Analyses were restricted to whole-blood RNA, without parallel assessment of protein levels. Although *CHI3L1* mRNA and YKL-40 protein have been shown to correlate in other disease contexts (Kazakova et al., 2014; Tanwar et al., 2002), direct validation in matched plasma/serum samples will be essential. Because *CHI3L1* is predominantly expressed by myeloid cells, whole-blood measurements cannot distinguish transcriptional regulation from shifts in leukocyte composition. Without leukocyte differentials or proportional cell estimates, the relative contribution of cell-type abundance versus gene-level activation cannot be determined. This limitation is common in peripheral biomarker studies and underscores the value of future work incorporating cell-type-specific resolution (e.g., immunophenotyping or single-cell RNA sequencing). Moreover, the cross-sectional design precludes conclusions about temporal dynamics or causal relationships.

All statistical analyses were based on ΔCt values, with fold-change transformations used exclusively for visualization. Technical factors such as RNA integrity, amplification efficiency, and reference-gene behavior can introduce modest variability, though intra-assay variability was low and ΔCt normalization minimized plate-to-plate drift. Although RIN values showed moderate variability, no significant associations with Ct values were detected, supporting the suitability of all samples for qPCR analyses. Nevertheless, residual effects on amplification efficiency cannot be ruled out. Reference genes were stable with respect to age and *APOE* genotype. Of note, *RNase P* showed modest cohort-wise variation, however this did not influence any conclusions, as *TREM2*-related analyses remained unchanged with alternative normalization. Still, the modest instability of *RNase P* between HC and AD (*p* = 0.0016) indicates that these null findings should be interpreted with caution, as reference-gene variability could mask weak associations. Some of the remaining variability in *CHI3L1* expression is therefore likely biological in origin and may reflect genuine heterogeneity in peripheral immune activation and *APOE*-dependent responses.

Finally, although several correlated transcripts (e.g., *MMP9*, *LRP1*) relate to vascular and BBB function, vascular outcomes were not assessed. Given, that *APOE* ε4 is associated with increased cerebrovascular vulnerability and higher ARIA incidence under anti-amyloid therapy (Honig et al., 2023; Van Dyck et al., 2023), peripheral inflammatory tone might conceptionally intersect with mechanisms influencing vascular response to treatment. However, this potential connection is speculative, and our data provide no evidence for predictive or causal relevance. Future studies specifically designed to examine peripheral immune signatures in treatment-exposed cohorts will be required to test this hypothesis.

## Conclusion

Our exploratory findings suggests that peripheral *CHI3L1* expression reflects *APOE* ε4-linked immune activity, which points toward higher expression in EOAD and in female ε4 carriers. These data support the concept of genotype- and sex-dependent systemic inflammatory phenotypes in AD. Validation in larger, longitudinal, and mechanistically resolved cohorts will be essential to define the clinical and biological relevance of peripheral *CHI3L1* in AD.

## Data Availability

The datasets generated for this study contain sensitive participant information and cannot be made publicly available due to ethical and legal restrictions. Requests to access the anonymized data should be directed to the corresponding author, and will be granted to qualified researchers in accordance with institutional and ethical guidelines.

## Glossary

AAO: Age at onset
AD: Alzheimer’s disease
APOE: Apolipoprotein E
APOE ε4: Apolipoprotein E epsilon 4 allele
ARIA: Amyloid-related imaging abnormalities
Aβ: Amyloid-beta
BBB: Blood–brain barrier
CAA: Cerebral amyloid angiopathy
cDNA: Complementary deoxyribonucleic acid
CHI3L1: Chitinase-3-like protein 1
CNS: Central nervous system
CSF: Cerebrospinal fluid
ΔCt: Delta cycle threshold
DNA: Deoxyribonucleic acid
EDTA: Ethylenediaminetetraacetic acid
EO: Early-onset
EOAD: Early-onset Alzheimer’s disease
EOMCI: Early-onset mild cognitive impairment
GAPDH: Glyceraldehyde 3-phosphate dehydrogenase
HC: Healthy control
IATI: Innotest Amyloid Tau Index
IL1B: Interleukin 1 beta
IQR: Interquartile range
LOAD: Late-onset Alzheimer’s disease
LO: Late-onset
LOMCI: Late-onset mild cognitive impairment
LRP1: Low-density lipoprotein receptor-related protein 1
MCI: Mild cognitive impairment
MMP9: Matrix metalloproteinase 9
MRI: Magnetic resonance imaging
mRNA: Messenger ribonucleic acid
PET: Positron emission tomography
qPCR: Quantitative polymerase chain reaction
RNA: Ribonucleic acid
RT-qPCR: Reverse transcription quantitative polymerase chain reaction
SNP: Single nucleotide polymorphism
TREM2: Triggering receptor expressed on myeloid cells 2
TNF: Tumor necrosis factor
YKL-40: Alternative name for CHI3L1

## References

[ref1] Albert M. S. DeKosky S. T. Dickson D. Dubois B. Feldman H. H. Fox N. C. . (2011). The diagnosis of mild cognitive impairment due to Alzheimer’s disease: recommendations from the National Institute on Aging-Alzheimer’s association workgroups on diagnostic guidelines for Alzheimer’s disease. Alzheimers Dement. 7, 270–279. doi: 10.1016/j.jalz.2011.03.008, 21514249 PMC3312027

[ref3] Antonell A. Mansilla A. Rami L. Lladó A. Iranzo A. Olives J. . (2014). Cerebrospinal fluid level of YKL-40 protein in preclinical and prodromal Alzheimer’s disease. J Alzheimer's Dis 42, 901–908. doi: 10.3233/JAD-140624, 25024322

[ref4] Bonneh-Barkay D. Wang G. Starkey A. Hamilton R. L. Wiley C. A. (2010). In vivo CHI3L1 (YKL-40) expression in astrocytes in acute and chronic neurological diseases. J. Neuroinflammation 7:34. doi: 10.1186/1742-2094-7-34, 20540736 PMC2892443

[ref5] Choi J. Lee H. W. Suk K. (2011). Plasma level of chitinase 3-like 1 protein increases in patients with early Alzheimer’s disease. J. Neurol. 258, 2181–2185. doi: 10.1007/s00415-011-6087-9, 21562723

[ref7] Deng Y. Li G. Chang D. Su X. (2020). YKL-40 as a novel biomarker in cardio-metabolic disorders and inflammatory diseases. Clin. Chim. Acta 511, 40–46. doi: 10.1016/j.cca.2020.09.035, 33002471

[ref8] Gispert J. D. Monté G. C. Suárez-Calvet M. Falcon C. Tucholka A. Rojas S. . (2017). The APOE ε4 genotype modulates CSF YKL-40 levels and their structural brain correlates in the continuum of Alzheimer’s disease but not those of sTREM2. Alzheimers Dement. Diagn. Assess. Dis. Monit. 6, 50–59. doi: 10.1016/j.dadm.2016.12.002, 28149943 PMC5266482

[ref9] Goldman J. S. Farmer J. M. Wood E. M. Johnson J. K. Boxer A. Neuhaus J. . (2005). Comparison of family histories in FTLD subtypes and related tauopathies. Neurology 65, 1817–1819. doi: 10.1212/01.wnl.0000187068.92184.63, 16344531

[ref10] Heneka M. T. Gauthier S. Chandekar S. A. Hviid Hahn-Pedersen J. Bentsen M. A. Zetterberg H. (2025). Neuroinflammatory fluid biomarkers in patients with Alzheimer’s disease: a systematic literature review. Mol. Psychiatry 30, 2783–2798. doi: 10.1038/s41380-025-02939-9, 40050444 PMC12092255

[ref12] Honig L. S. Barakos J. Dhadda S. Kanekiyo M. Reyderman L. Irizarry M. . (2023). ARIA in patients treated with lecanemab (BAN2401) in a phase 2 study in early Alzheimer’s disease. Alzheimers Dement. 9:e12377. doi: 10.1002/trc2.12377, 36949897 PMC10026083

[ref13] Hu Y. Hu X. d. He Z. q. Liu Y. Gui Y. k. Zhu S. h. . (2024). Anesthesia/surgery activate MMP9 leading to blood-brain barrier disruption, triggering neuroinflammation and POD-like behavior in aged mice. Int. Immunopharmacol. 135:112290. doi: 10.1016/j.intimp.2024.112290, 38796964

[ref14] Jack C. R. Bennett D. A. Blennow K. Carrillo M. C. Dunn B. Haeberlein S. B. . (2018). NIA-AA research framework: toward a biological definition of Alzheimer’s disease. Alzheimers Dement. 14, 535–562. doi: 10.1016/j.jalz.2018.02.018, 29653606 PMC5958625

[ref15] Kazakova M. H. Staneva D. N. Koev I. G. Staikov D. G. Mateva N. Timonov P. T. . (2014). Protein and mRNA levels of YKL-40 in high-grade glioma. Folia Biol. (Praha) 60, 261–267. doi: 10.14712/fb2014060060261, 25629266

[ref16] Kester M. I. Teunissen C. E. Sutphen C. Herries E. M. Ladenson J. H. Xiong C. . (2015). Cerebrospinal fluid VILIP-1 and YKL-40, candidate biomarkers to diagnose, predict and monitor Alzheimer’s disease in a memory clinic cohort. Alzheimer's Res Ther 7:59. doi: 10.1186/s13195-015-0142-1, 26383836 PMC4574487

[ref17] Kloske C. M. Wilcock D. M. (2020). The important interface between apolipoprotein E and neuroinflammation in Alzheimer’s disease. Front. Immunol. 11:754. doi: 10.3389/fimmu.2020.00754, 32425941 PMC7203730

[ref18] Koriath C. Kenny J. Adamson G. Druyeh R. Taylor W. Beck J. . (2020). Predictors for a dementia gene mutation based on gene-panel next-generation sequencing of a large dementia referral series. Mol. Psychiatry 25, 3399–3412. doi: 10.1038/s41380-018-0224-0, 30279455 PMC6330090

[ref19] Kühner C. Bürger C. Keller F. Hautzinger M. (2007). Reliabilität und Validität des revidierten Beck-Depressionsinventars (BDI-II). Nervenarzt 78, 651–656. doi: 10.1007/s00115-006-2098-7, 16832698

[ref20] Lehrner J. Kogler S. Lamm C. Moser D. Klug S. Pusswald G. . (2015). Awareness of memory deficits in subjective cognitive decline, mild cognitive impairment, Alzheimer’s disease and Parkinson’s disease. Int. Psychogeriatr. 27, 357–366. doi: 10.1017/S1041610214002245, 25382659

[ref21] Llorens F. Thüne K. Tahir W. Kanata E. Diaz-Lucena D. Xanthopoulos K. . (2017). YKL-40 in the brain and cerebrospinal fluid of neurodegenerative dementias. Mol. Neurodegener. 12:83. doi: 10.1186/s13024-017-0226-4, 29126445 PMC5681777

[ref22] Montagne A. Zhao Z. Zlokovic B. V. (2017). Alzheimer’s disease: a matter of blood-brain barrier dysfunction? J. Exp. Med. 214, 3151–3169. doi: 10.1084/jem.20171406, 29061693 PMC5679168

[ref23] Nee L. E. McMorrow T. Campbell E. Slattery C. Ryan M. P. (2004). TNF-alpha and IL-1beta-mediated regulation of MMP-9 and TIMP-1 in renal proximal tubular cells. Kidney Int. 66, 1376–1386. doi: 10.1111/j.1523-1755.2004.00900.x, 15458430

[ref24] Nichols E. Steinmetz J. D. Vollset S. E. Fukutaki K. Chalek J. Abd-Allah F. . (2022). Estimation of the global prevalence of dementia in 2019 and forecasted prevalence in 2050: an analysis for the global burden of disease study 2019. Lancet Public Health 7, e105–e125. doi: 10.1016/S2468-2667(21)00249-8, 34998485 PMC8810394

[ref25] Pase M. P. Himali J. J. Puerta R. Beiser A. S. Gonzales M. M. Satizabal C. L. . (2024). Association of Plasma YKL-40 with MRI, CSF, and cognitive markers of brain health and dementia. Neurology 102:e208075. doi: 10.1212/WNL.0000000000208075, 38290090 PMC11383876

[ref26] Philippe C. Haeusler D. Mitterhauser M. Ungersboeck J. Viernstein H. Dudczak R. . (2011). Optimization of the radiosynthesis of the Alzheimer tracer 2-(4-N-[11C]methylaminophenyl)-6-hydroxybenzothiazole ([11C]PIB). Appl. Radiat. Isot. 69, 1212–1217. doi: 10.1016/j.apradiso.2011.04.010, 21550258

[ref27] Pusswald G. Moser D. Gleiss A. Janzek-Hawlat S. Auff E. Dal-Bianco P. . (2013). Prevalence of mild cognitive impairment subtypes in patients attending a memory outpatient clinic--comparison of two modes of mild cognitive impairment classification. Results of the Vienna conversion to dementia study. Alzheimers Dement. 9, 366–376. doi: 10.1016/j.jalz.2011.12.009, 23164551

[ref28] Sampedro F. Vilaplana E. de Leon M. J. Alcolea D. Pegueroles J. Montal V. . (2015). APOE-by-sex interactions on brain structure and metabolism in healthy elderly controls. Oncotarget 6, 26663–26674. doi: 10.18632/oncotarget.5185, 26397226 PMC4694943

[ref29] Shackleton B. Ringland C. Abdullah L. Mullan M. Crawford F. Bachmeier C. (2019). Influence of matrix metallopeptidase 9 on Beta-amyloid elimination across the blood-brain barrier. Mol. Neurobiol. 56, 8296–8305. doi: 10.1007/s12035-019-01672-z, 31209784 PMC6842100

[ref31] Sun N. Victor M. B. Park Y. P. Xiong X. Scannail A. N. Leary N. . (2023). Human microglial state dynamics in Alzheimer’s disease progression. Cell 186, 4386–4403.e29. doi: 10.1016/j.cell.2023.08.037, 37774678 PMC10644954

[ref32] Tabaraud F. Leman J. P. Milor A. M. Roussie J. M. Barrière G. Tartary M. . (2012). Alzheimer CSF biomarkers in routine clinical setting. Acta Neurol. Scand. 125, 416–423. doi: 10.1111/j.1600-0404.2011.01592.x, 21954973

[ref33] Tanwar M. K. Gilbert M. R. Holland E. C. (2002). Gene expression microarray analysis reveals YKL-40 to be a potential serum marker for malignant character in human glioma1. Cancer Res. 62, 4364–4368, 12154041

[ref34] Tondo G. Iaccarino L. Caminiti S. P. Presotto L. Santangelo R. Iannaccone S. . (2020). The combined effects of microglia activation and brain glucose hypometabolism in early-onset Alzheimer’s disease. Alzheimer's Res Ther 12:50. doi: 10.1186/s13195-020-00619-0, 32354345 PMC7193377

[ref35] Van Dyck C. H. Swanson C. J. Aisen P. Bateman R. J. Chen C. Gee M. . (2023). Lecanemab in early Alzheimer’s disease. N. Engl. J. Med. 388, 9–21. doi: 10.1056/NEJMoa2212948, 36449413

[ref36] Van Gool B. Storck S. E. Reekmans S. M. Lechat B. Gordts P. L. S. M. Pradier L. . (2019). LRP1 has a predominant role in production over clearance of aβ in a mouse model of Alzheimer’s disease. Mol. Neurobiol. 56, 7234–7245. doi: 10.1007/s12035-019-1594-2, 31004319 PMC6728278

[ref37] Vanderstichele H. Van Kerschaver E. Hesse C. Davidsson P. Buyse M. A. Andreasen N. . (2000). Standardization of measurement of beta-amyloid(1-42) in cerebrospinal fluid and plasma. Amyloid 7, 245–258. doi: 10.3109/13506120009146438, 11132093

[ref38] Vanmechelen E. Vanderstichele H. Davidsson P. Van Kerschaver E. Van Der Perre B. Sjögren M. . (2000). Quantification of tau phosphorylated at threonine 181 in human cerebrospinal fluid: a sandwich ELISA with a synthetic phosphopeptide for standardization. Neurosci. Lett. 285, 49–52. doi: 10.1016/S0304-3940(00)01036-3, 10788705

[ref39] Walker K. A. Ficek B. N. Westbrook R. (2019). Understanding the role of systemic inflammation in Alzheimer’s disease. ACS Chem. Neurosci. 10, 3340–3342. doi: 10.1021/acschemneuro.9b00333, 31241312

[ref41] Xu F. Xu J. Wang Q. Gao F. Fu J. Yan T. . (2024). Serum YKL-40 as a predictive biomarker of cerebral amyloid Angiopathy-related intracerebral hemorrhage recurrence. J Alzheimer's Dis 99, 503–511. doi: 10.3233/JAD-231125, 38669531

[ref9001] Zhang W. Zhou X. Yin J. Zhao W. Huang C. Zhang C. . (2023). YKL-40 as a novel biomarker related to white matter damage and cognitive impairment in patients with cerebral small vessel disease. Brain Res. 1807:148318. doi: 10.1016/j.brainres.2023.148318 36898474

