# Supplementary material for: Peripheral CHI3L1 expression is associated with APOE ε4 status in early-onset Alzheimer’s disease
Source: Front Aging Neurosci. 2025 Dec 4;17:1730319. doi: 10.3389/fnagi.2025.1730319 (PMC12711778; doi:10.3389/fnagi.2025.1730319)
Supplement: Supplementary file 1 [file Data_Sheet_1.docx]

**
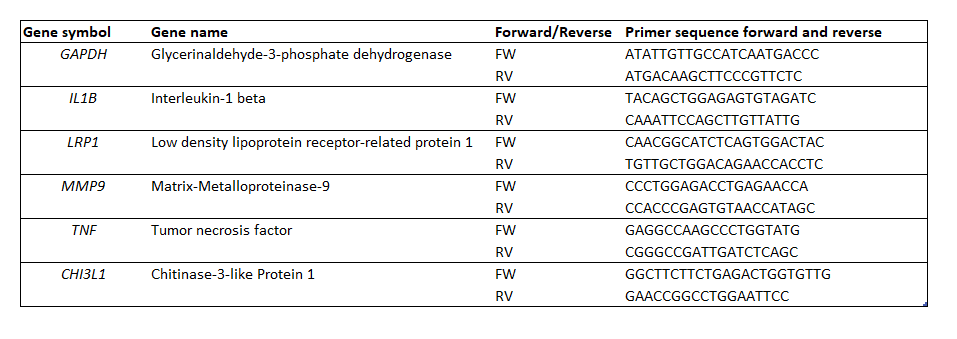
Supplementary Table 1 - Sequences of primers used for SYBR green-based qPCR assays**

**
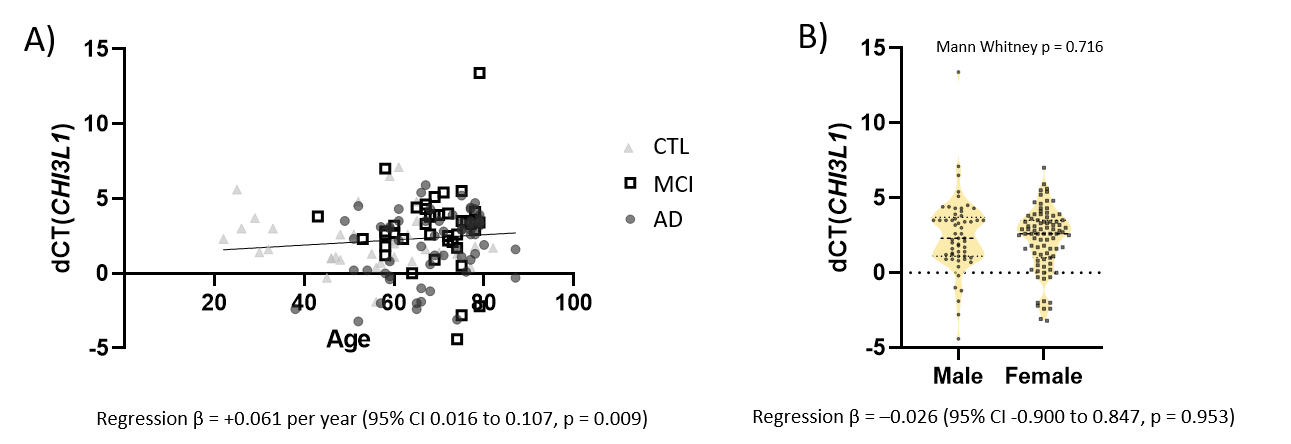
**

**Supplementary Figure 1 - Relationship of *CHI3L1* ΔCt with age and sex.**

(A) Scatter plot of *CHI3L1* ΔCt values versus age at blood draw across HC, MCI, and AD participants. Each point represents an individual; symbol shapes denote diagnostic groups. (B) Distribution of *CHI3L1* ΔCt values by sex (pooled across all participants). Individual datapoints are shown with violin/box overlays. No sex effect was observed (Mann-Whitney p = 0.716). Lower ΔCt values correspond to relatively higher transcript abundance.

**
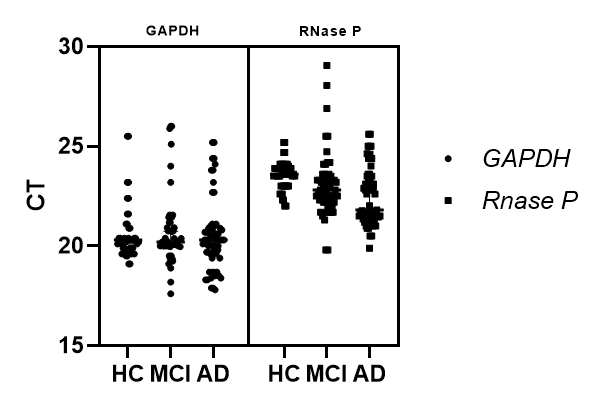
**

**Supplementary Figure 2. Distribution of *GAPDH* and *RNase P* Ct values across diagnostic groups.** Scatterplots showing the distribution of raw Ct values for *GAPDH* (left) and *RNase P* (right) in healthy controls (HC), mild cognitive impairment (MCI), and Alzheimer’s disease (AD). *GAPDH* displayed stable Ct values with no significant group differences. RNase P showed a modest HC-AD difference in line with Kruskal-Wallis/Dunn results. Both genes exhibited narrow distributions and low coefficients of variation, supporting their use as reference genes for ΔCt normalization.

**
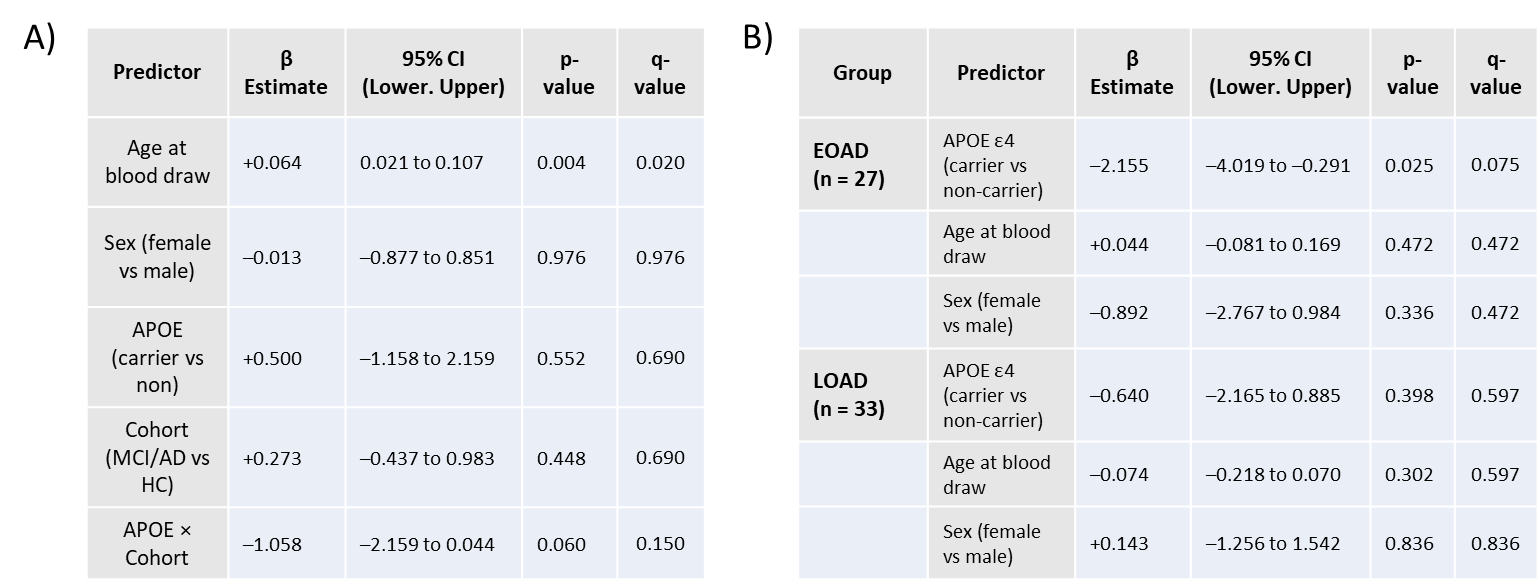
**

**(**A) Regression model including HC, MCI, and AD participants (corresponding to Figure 2A). Predictors were *APOE* ε4 carrier status. cohort (HC, MCI, AD), age at blood draw, sex, and an APOE × cohort interaction term. (B) Regression models stratified by AAO within AD patients (corresponding to Figure 2B). Separate models were run for EOAD and LOAD with predictors *APOE* ε4 carrier status, age at blood draw, and sex. In all models the dependent variable was *CHI3L1* ΔCt. Negative β values indicate lower ΔCt (higher transcript abundance) in *APOE* ε4 carriers compared to non-carriers. Abbreviations: HC, healthy controls; MCI, mild cognitive impairment; AD, Alzheimer’s disease; EOAD, early-onset Alzheimer’s disease; LOAD, late-onset Alzheimer’s disease; APOE ε4, apolipoprotein E epsilon 4 allele; ΔCt, delta cycle threshold; CI, confidence interval; β, regression coefficient.

**Supplementary Table 2 – Multivariable regression analyses of *CHI3L1* ΔCt values corresponding to Figure 2.**

**
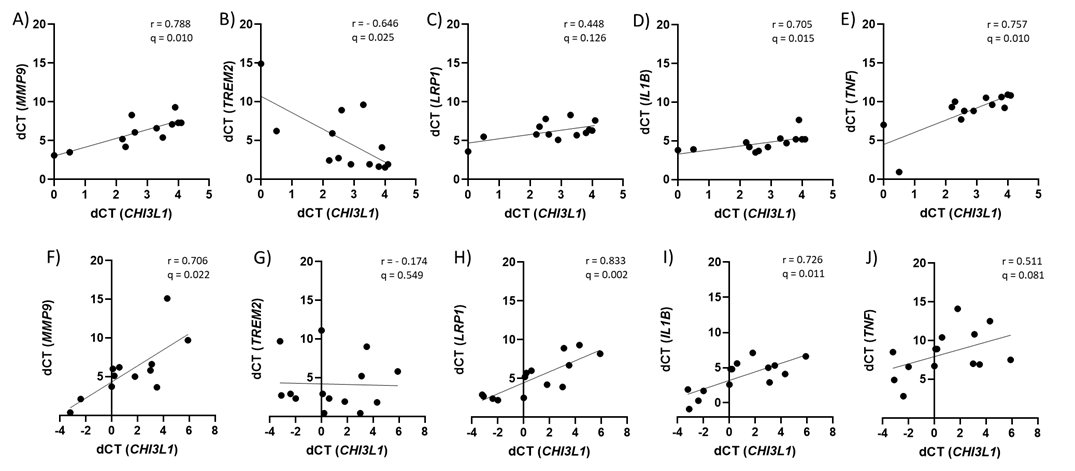
**

**Supplementary Figure 3 - Correlations between peripheral CHI3L1 expression and inflammatory markers in female APOE ε4+ MCI and AD Patients**

(A–E) Correlations between CHI3L1 ΔCt values and inflammatory markers (*IL1B*, *TNF*, *LRP1*, *TREM2*, *MMP*9) in female APOE ε4+ MCI. (F–J) Correlations between CHI3L1 dCt values and inflammatory markers (*IL1B*, *TNF*, *LRP1*, *TREM2*, *MMP9*) in female APOE ε4+ AD. Lower ΔCt values indicate higher CHI3L1 expression. Spearman correlation coefficients (r) and q-values are shown (FDR adjusted); q-values < 0.05 were considered statistically significant. Abbreviations: MCI, mild cognitive impairment; AD, Alzheimer’s disease; APOE ε4+, APOE ε4 carriers; IL1B, interleukin-1 beta; TNF, tumor necrosis factor; LRP1, low-density lipoprotein receptor-related protein 1; TREM2, triggering receptor expressed on myeloid cells 2; MMP9, matrix metalloproteinase 9; CHI3L1, chitinase-3-like protein.

**Supplementary Table 3 – Overview of correlation of *CHI3L1* with inflammatory markers stratified by AAO and sex**

Spearman’s correlation coefficients (r) are shown together with unadjusted p-values; Benjamini–Hochberg false discovery rate (FDR) was used for multiple testing and significance levels (significance threshold q < 0.05). Analyses were stratified by diagnostic subgroup (EOMCI, LOMCI, EOAD, LOAD) and sex (male, female). Significant correlations are highlighted in bold. Abbreviations: EOMCI, early-onset mild cognitive impairment; LOMCI, late-onset mild cognitive impairment; EOAD, early-onset Alzheimer’s disease; LOAD, late-onset Alzheimer’s disease; IL1B, interleukin 1 beta; TNF, tumor necrosis factor; LRP1, low-density lipoprotein receptor-related protein 1; TREM2, triggering receptor expressed on myeloid cells 2; MMP9, matrix metallopeptidase 9; ns, not significant.

**Supplementary Table 4 - Associations between peripheral *CHI3L1* expression and systemic inflammatory/metabolic markers**

Spearman’s correlation coefficients (r) are shown together with unadjusted p values. Benjamini–Hochberg false discovery rate (FDR) was used for multiple testing and significance levels (significance threshold q < 0.05). Significant correlations are highlighted in bold. Abbreviations: HC, healthy controls; MCI, mild cognitive impairment; EOMCI, early-onset mild cognitive impairment; LOMCI, late-onset mild cognitive impairment; AD, Alzheimer’s disease; EOAD, early-onset Alzheimer’s disease; LOAD, late-onset Alzheimer’s disease; CRP, C-reactive protein; Chol., total cholesterol; HDL, high-density lipoprotein; LDL, low-density lipoprotein; ns, not significant.
